# Long Term Physical Activity Improves Quality of Life Perception, Healthy Nutrition, and Daily Life Management in Elderly: A Randomized Controlled Trial

**DOI:** 10.3390/nu14122527

**Published:** 2022-06-17

**Authors:** Giovanni Fiorilli, Andrea Buonsenso, Marco Centorbi, Giuseppe Calcagno, Enzo Iuliano, Antonella Angiolillo, Santina Ciccotelli, Alessandra di Cagno, Alfonso Di Costanzo

**Affiliations:** 1 Department of Medicine and Health Sciences, University of Molise, 86100 Campobasso, Italy; fiorilli@unimol.it (G.F.); andrea.buonsenso@unimol.it (A.B.); marco.centorbi@hotmail.it (M.C.); giuseppe.calcagno@unimol.it (G.C.); 2 Faculty of Psychology, eCampus University, 22060 Novedrate, Italy; enzo.iuliano@uniecampus.it; 3 Centre for Research and Training in Medicine of Aging, Department of Medicine and Health Science “V. Tiberio”, University of Molise, 86100 Campobasso, Italy; angiolillo@unimol.it (A.A.); santinaciccotelli@libero.it (S.C.); alfonso.dicostanzo@unimol.it (A.D.C.); 4 Department of Motor, Human and Health Sciences, University of Rome “Foro Italico”, 00197 Rome, Italy

**Keywords:** exercise, diet, wellbeing, aging, daily life management

## Abstract

Physical activity (PA) is a key element in the management of successful aging. The aim of this paper was to show the effects of PA on the quality of life perception, nutritional status, and daily life management of 178 older adults (aged 63.87 ± 8.17) randomly assigned to an Experimental Group (EG), which performed moderate-to-high intensity aerobic and strengthening training, and a Control Group (CG) which performed low-impact PA, assessed after 6, 12, and 24 months. The Short-Form Health Survey (SF-36), Mini Nutritional Assessment (MNA), and Physical Activity Scale for the Elderly (PASE) were used for the study. In the SF-36 assessment, EG showed a good quality of life perception maintained after 24 months, while CG showed a worsening in the same period (*p* = 0.018). The EG reported a significant better nutritional status as compared to pre-intervention assessment (*p* = 0.003) and to CG (*p* < 0.001). Regarding the PASE, the EG showed a higher level of weekly activities than the CG after 24 months (*p* = 0.011), while the CG showed a worsening after 12 months (*p* = 0.008). The prolonged engagement in moderate-to high-intensity PA allowed the maintenance of a good quality of life perception, a good level of nutritional status, and daily life activities.

## 1. Introduction

Quality of life is a multidimensional and broad concept closely linked to the concept of both the physical and psychological health of individuals and their community [1]. Age is a non-modifiable risk factor during which the worsening of functional capacities and the increase in the diseases’ risk is an expected and natural phenomenon [2]. The results of previous studies showed that regular long-term physical activity (PA) prevents the occurrence of several diseases in the elderly, and there is a general consensus that PA plays a key role in promoting a “successful ageing” [2]. This concept not only includes avoiding diseases and disabilities but also promotes a healthy lifestyle in terms of maintaining good physical and cognitive abilities and being involved in daily life activities with a willing acceptance of the conditions related to old age [3]. The WHO’s ‘Global Recommendations on Physical Activity for Health’ state that “adults 65 and older should engage in 150 min of moderate- or 75 min of vigorous-intensity aerobic activity and two or more days of muscle-strengthening activity per week” [4]. Maintaining correct healthy habits throughout life allows for successful aging. Main lifestyle factors include the number of calories ingested and the composition and quality of diet, physical exercise, active social contacts, social communication, and avoiding stressful situations [5]. The potential protective role of an active lifestyle could be obtained both by an increase in household activity and engagement in recreational and sportive activities by older people [6]. Ngandu et al. [7] showed that multi-intervention programs based on diet, exercise, and social interaction promotion, could improve or maintain cognitive functioning and good quality of life. Moreover, gradual multi-sensory deterioration, by providing less stimulation to various areas of the brain, accelerates the decline in the elderly. Environmental factors linked to active lifestyles seem to positively influence the neurodegenerative processes, promoting adherence and compliance and, consequently, improving the quality of life perception (QoLP) [8]. Conversely, inactivity, loneliness, and depression increase the risk of falling into discomfort and illnesses [9].

An epidemiological analysis of the European population highlighted a systematic increase in the percentage of older adults within the total population, correlated to the lengthening of life expectancy thanks to medical progress. The percentage of individual decline increases with age and is associated with decreased health and quality of life [10]. The QoLP, in terms of the perception of one’s own health and reacting to the factors related to the loss of efficiency, has become a key point of view in therapeutic intervention, with prognostic significance [11,12]. The WHO provides the definition of the QoLP as: “An individual’s perception of their position in life, in the context of the culture in which they live and in relation to their goals, expectations, standards and concerns” [13]. Moreover, perceived general health, which determines a good quality of life in older adults, is not only associated with an acceptable physical efficiency but also with a healthy nutritional status [14]. A proper nutritional plan ensures health and improves the quality of life in the elderly, considering that older adults are at risk of undernutrition [15]. It is well known that undernutrition can lead to hormonal disorders even in young people [16]. Therefore, weight loss and pessimism in the future are clear symptoms of a worse QoLP in the elderly [17]. Most previous studies aimed to evaluate the effects of PA on QoLP, both the level and typology of the PA performed by the participants were assessed using questionnaires [18], while in the present study, PA was administered and closely monitored directly by operators.

The aim of this intervention study was to demonstrate that a 24-month multimodal PA program, performed by an older adult group (experimental group), improves the QoLP, nutritional status, and daily life management more than a low-impact PA with the same duration and frequency performed by a control group. We hypothesized that choosing a healthy, active lifestyle induced by a long-term period of moderate-to-high PA may lead to better life satisfaction, promoting good practices and, consequently, successful aging.

## 2. Materials and Methods

This was a two-year study, designed as a randomized controlled trial, aimed to evaluate the long-term effects of PA on QoLP, nutritional status, and daily life management in older adults. The study has been approved by the Ethics Committee of University of Molise (26119_II/1).

### 2.1. Participants

**Figure 1:**
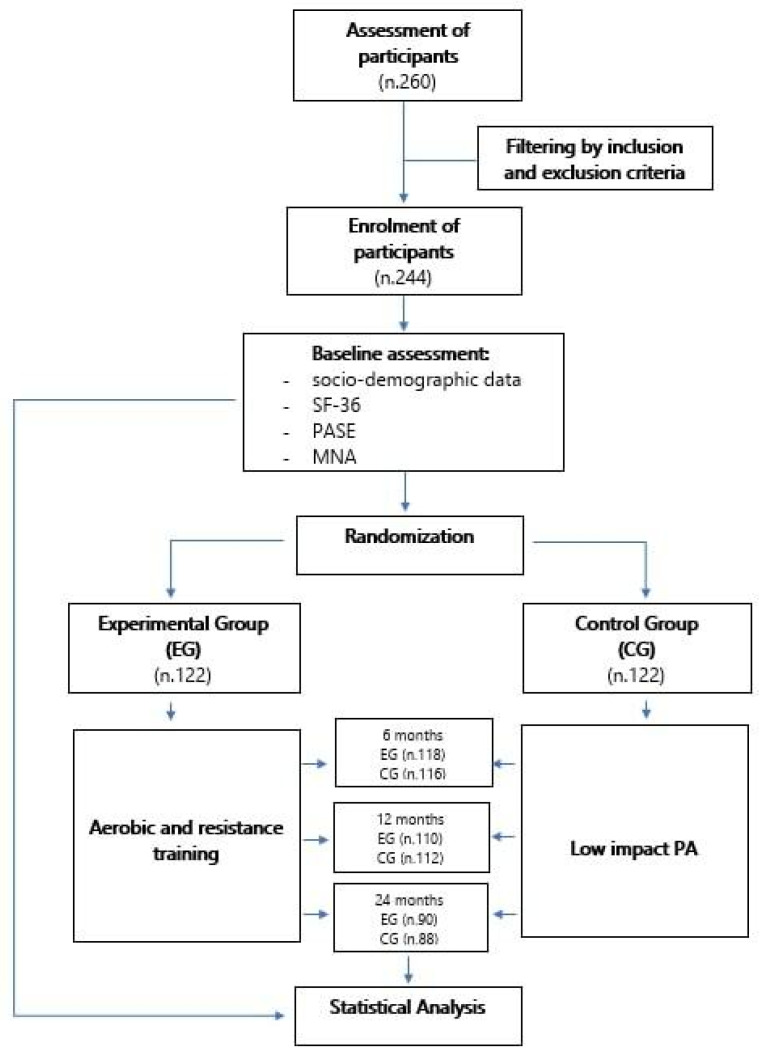
Flowchart of the study.

Two hundred and sixty subjects were recruited from the Centre for Research and Training from the Medicine of Ageing of the University of Molise, but only 244 (age 63.87 ± 8.17) met the inclusion criteria and were randomly assigned to the Experimental Group (EG) (*n* = 122; age 62.12 ± 7.88) or the Control Group (CG) (*n* = 122; age 65.64 ± 8.12). After randomization, the two groups were not significantly different in age. The inclusion criteria were selected as those aged ≥ 50 years who have a sedentary or normally-active lifestyle, according to the definition of Pate et al. [19]. The exclusion criteria were: (a) medical conditions presenting contraindications to the practice of PA; (b) an inability to walk for 6 min (6-min walking—test) [20]; (c) regular participation in structured PA programs of moderate/vigorous intensity. One-hundred-seventy-eight participants, 90 in the EG and 88 in the CG, completed the 24-month intervention and were included in the analysis. The sample characteristics are shown in Table 1. All of the subjects were authorized by medical certification to practice PA. The flowchart of the study design is illustrated in Figure 1. This study was designed and conducted in accordance with the principles of the Declaration of Helsinki, and all of the participants provided written informed consent.

### 2.2. Procedures

The perceived quality of life, the nutritional status, and the weekly level of PA were assessed through the Short-Form 36-item health survey (SF-36) [21], the Mini-Nutritional Assessment (MNA) [22], and the Physical Activity Scale for the Elderly (PASE) [23], respectively, before the intervention, and at 6, 12, and 24 months.

After the baseline assessments, each subject was identified by a progressive number and randomly assigned in a ratio of 1:1 to the experimental or control group, using a list of random numbers generated by statistical software (SPSS). The list was kept in a sealed envelope, and a researcher, not directly involved in the recruitment and the evaluation of the participants, assigned the allocation numbers.

#### 2.2.1. Short Form 36-Item

The Short-Form 36-item questionnaire measures 8 health-related parameters: physical functioning (10 items), limitations in daily activities due to emotional problems (3 items) and physical problems (4 items), social functioning (2 items), mental health (5 items), body pain (2 items), vitality (4 items), general health perception (5 items), and a single question on the change in health status during the last year. The SF-36 questionnaire can be self-rating. All of the questions refer to a period of four weeks before the completion of the questionnaire and is aimed at assessing the quality of life and health status. The scores are converted to a standardized score scale from 0 to 100, with higher scores indicating better health. The SF-36 has a good test–retest reliability (0.70) [24].

#### 2.2.2. Mini Nutritional Assessment (MNA)

The MNA assessment is designed for a simple and rapid evaluation of the nutritional status of the elderly. The questionnaire includes 18 items divided into 4 domains: anthropometric assessment (weight, height, BMI, and arm and calf circumferences), global assessment (lifestyle, medication, and mobility), dietary assessment (number of meals and food and fluid intake), and subjective perception of health and nutrition. Each item has a numerical value, and the total score has a maximum value of 30. The MNA classifies the nutritional status as adequate (score from 24 to 30), at risk of malnutrition (score from 17 to 23.5), and poor (score less than 17). The MNA has a good test–retest reliability (0.89) [25].

#### 2.2.3. Physical Activity Scale for the Elderly (PASE)

This questionnaire evaluates the PA performed during the previous week and consists of 12 items that are self-administered. The questionnaire analyses 3 types of PA: leisure time, household, and work activities. The score ranged from 0 to 793; a higher score indicates a greater level of weekly PA [26]. The PASE has a test–retest reliability of 0.75 [27].

### 2.3. Intervention

In the 24-month exercise program, the EG performed 3 sessions per week for 1 h each. The exercise program was individualized based on physical function, physical fitness level, health status, and exercise responses. The exercise intensity was measured according to Borg’s Rating of Perceived Exertion (RPE), a 6-to-20 points scale: a score of <11 is considered an effort equal to light exercise, 12–13 a moderate exercise, and 14–17 a vigorous exercise [28]. Each training session consisted of a warm-up, a central phase, and a cool-down phase. The warm-up phase lasted 10 min and consisted of light-to-moderate intensity (RPE 10–12) aerobic activity. During this phase, walking/jogging activities were used to activate the biggest muscular groups. The central phase of the training session consisted of aerobic and resistance exercises: aerobic exercises were performed for 15–20 min using a treadmill, stationary bike, arm-cycle ergometer, and/or running; the duration and intensity of the exercise were gradual over time, starting with multiple bouts (<2 min) of light-to-moderate intensity (RPE max 12–13) exercises, up to two 10-min bouts of moderate-to-vigorous exercise (RPE max 16–17) after several weeks of training; subsequently, resistance exercises were performed for 20–25 min, involving the main muscular groups of limbs and trunk; the resistance exercises were based on bodyweight and dumbbell exercises (up to 2 kg); the duration and intensity were progressive over time, starting with 2 series of 10 repetitions (RPE max 11–12) up to 6 series of 20 repetitions (RPE max 14–16), for each muscular group. To promote the required exertion and enjoyment for the performed activity, different music for each phase of the protocol was used to provide the executive rhythm (music beats) and to decrease the rating of perceived exertion (RPE) and subjective fatigue during the exercise [29]. The musical genre was chosen so that it could be pleasant and enjoyable for each subject. Finally, the cool-down phase, lasting 10 min, consisted of flexibility exercises for each major muscle/tendon group. The participants were invited to stretch to the point of feeling tightness or slight discomfort, hold a static stretch for 20–30 s and repeat the exercise 2–3 times for each muscle/tendon group.

In order to assure optimal supervision during the exercise, the participants were divided into subgroups, with a maximum of 12 participants. Experienced trainers managed all of the training sessions, providing the same program to each group. The subjects in the same group were encouraged to interact with each other and with the trainer, promoting social communication and stimulation. The training program based on the RPE system was chosen and designed to be easily reproducible at home to promote a physically active lifestyle after the end of the study.

The CG performed 3 sessions per week of 1 h, each one of low-impact PA, consisting of stretching, balance exercises, posture education, and coordination. The PA intensity was light (RPE < 11). The activity of the CG was supervised by qualified trainers in order to assure the effective intensity of the training and the correct execution of the exercises. The participation rate of each subject was >50%. The participants were forbidden to adhere to other training programs.

### 2.4. Statistical Analysis

All of the statistical analyses were carried out using SPSS statistics version 17.0 (IBM, Chicago, IL, USA) software. All of the variables were tested for a normal distribution using the Kolmogorov–Smirnov test. For all of the statistical tests, the α level was set to 0.05. The data are presented as mean and standard deviations. All of the variables (SF-36, MNA, PASE scores) showed a normal distribution, so the 3 separate RM-ANOVA were performed (one for each variable), with intervention (EG vs. CG) and time (pre-intervention vs. 6 months vs. 12 months vs. 24 months) as repeated factors. Post-hoc comparisons were performed using the Bonferroni test. Statistical analysis was performed on a total of 178 participants (90 EG and 88 CG). The power of the analyses achieved was computed using G*Power software v. 3.1.9.7. The calculation was performed considering a sample size = 178 participants and an α error probability = 0.95. The results of the computation indicated a power = 0.988.

## 3. Results

The statistical analysis showed that the EG and CG were homogenous at baseline.

**Figure 2:**
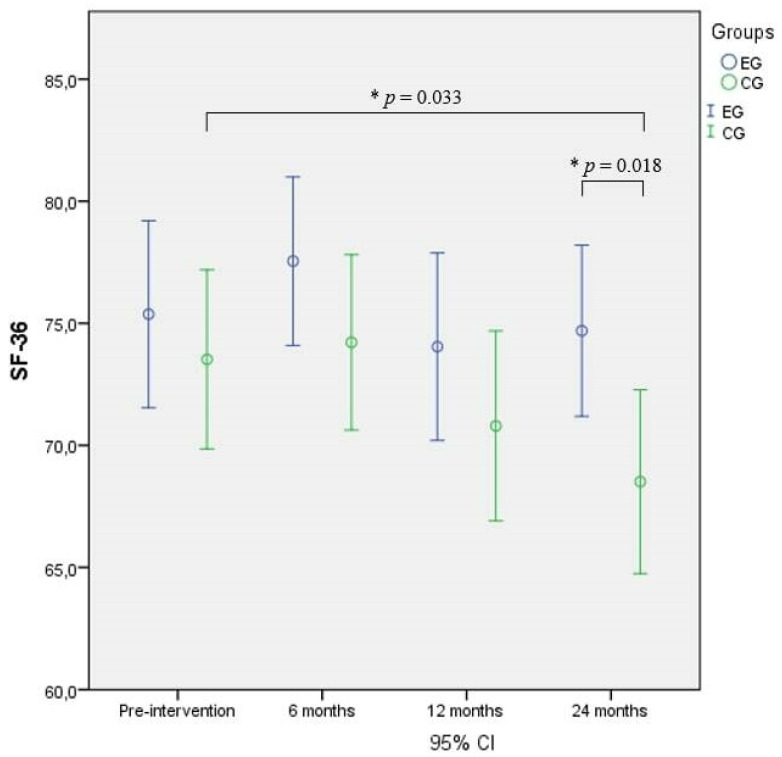
Short Form 36-item (SF-36) comparison between and within groups. * = Significant difference.

The RM-ANOVA performed on SF-36 showed a significant difference over time and between the groups, and the post-hoc test revealed significantly lower scores in the CG compared to the EG (*p* = 0.018) and to baseline (*p* = 0.033) after 24 months of intervention (Figure 2).

**Figure 3:**
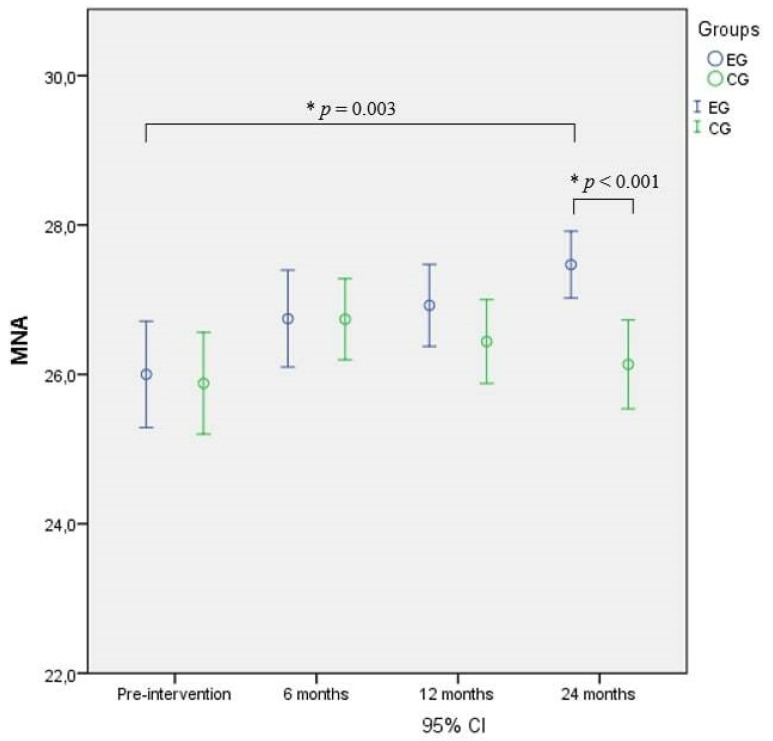
Mini nutritional assessment (MNA) comparison between and within groups. * = Significant difference.

The RM-ANOVA performed on the MNA showed significant differences over time between the groups and in the interaction, and the post-hoc test revealed significantly higher scores in the EG compared to the CG (*p* < 0.001) and to baseline (*p* = 0.003) after 24 months of intervention (Figure 3).

**Figure 4:**
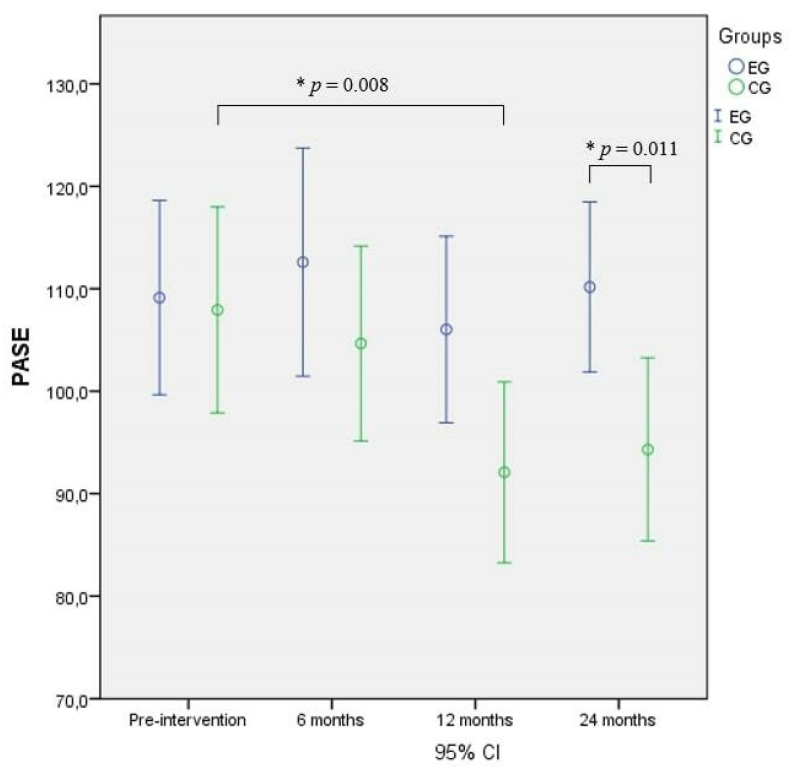
Physical activity scale for the elderly (PASE) comparison between and within groups. * = Significant difference.

The RM-ANOVA performed on PASE showed significant differences over time and between groups and in the interaction, and the post-hoc test revealed significantly higher scores in the EG compared to the CG (*p* = 0.011) after 24 months and to baseline (*p* = 0.008) after 12 months of intervention (Figure 4).

The results obtained by the two groups in the three tests are shown in Table 2.

## 4. Discussion

The main result of this study was that the EG group, engaged in long-term moderate-to-vigorous PA programs, was able to maintain a good QoLP up to two years of intervention despite their advancing age and also improved their nutritional behaviors with a cascading effect on activities in daily life. While the CG, who performed low-impact PA, significantly worsened their nutritional status and, over time, their QoLP, resulting in significantly less activity in their daily life activities. QoLP depends on overall life satisfaction due to good daily life conditions, maintaining behavioral competencies, and physical and physiological health [30]. The lowering sense of health in the elderly depends on the physiological process of aging but also on changing life conditions and the lack of subjective perception of any symptomatic improvement [31]. This condition could be counteracted by inclusion in different effective activities, such as PA programs, mostly through a prolonged engagement in regular exercise, which improves self-efficacy and, consequently, their self-esteem [32,33].

In the present study, the EG, after a 24-month period of moderate-to-high intensity PA programs, not only maintained their QoLP but also showed improved nutritional habits. Differently, the CG, who performed low-impact PA, significantly worsened their nutritional status, confirming the potential protective role of long-term-moderate-to-high intensity PA participation on several aspects of life [34]. It is well known that sedentarism and loneliness worsen nutritional status and that malnutrition is an early identifier of psychological problems in the elderly [35]. The link between PA and good nutrition allows older adults to be engaged in a correct and healthy lifestyle and consequently improve their QoLP.

Nutritional status in this study served as an indicator of the physical and psychological health of participants induced by the long-term protocol of the proposed PA.

Considering that the results of this study were achieved only after 24 months of PA, compliance and long-term adherence are mandatory for older adults to maintain a good perceived health-related quality of life. Stating this assumption, several recommendations for organizing PA programs should be taken into account, such as ensuring an optimal frequency and intensity of exercise, a wide variety of exercise modalities, and a proper degree of sociality.

Conflicting findings regarding the optimal intensity were previously shown in several studies [36,37,38]. However, regular PA practice performed at RPE between 12 and 17 involving the main muscular groups, such as in the present study, allowed for the maintenance of a good QoLP and significantly improved nutritional status. Moreover, sharing the PA challenges with subjects with similar characteristics and problems motivated the EG exercise participation and improved adherence [39]. The CG, working with an RPE of < 11, did not reach the same results.

The differences amongst the exercise modalities have proved to be essential for reaching different goals. Aerobic exercise is an effective treatment to improve quality of life and psychological wellbeing, promoting cardiovascular improvements, brain vascularization, and benefits to brain plasticity [40]. Resistance training improves functional impairments and functional status, such as strength and balance, increasing feelings of self-efficacy, self-perceptions of control, and reducing emotional stress [41]. Moreover, social contact during regular exercise, collectively performed, and even involving different generations may achieve positive feedback from other people and an increased sense of self-esteem [42,43]. Conversely, low-impact PA based on low-intensity programs did not allow for a sufficient rate of exertion to improve the sense of efficiency and counteract the worsening QoLP linked to aging.

The EG protocol allowed for the improvement of QoLP stimulation, at the same time, positive spillover effects on the daily life activities assessed by PASE. In fact, EG, during the intervention time, maintained their usual level of planned and unplanned activities in everyday life, such as leisure, household, recreational, and sports activities. In previous studies, different self-assessment questionnaires have been used to quantify the amount of daily PA carried out over a period of seven days [44,45]. PASE turned out to be extremely useful for these research purposes due to its ease of administration and accuracy, validated by comparing the questionnaire with a measurement of indirect and direct PA [23,24,25,26].

Regarding the nutritional status, it is well known that inactivity and loneliness induce the risk of malnutrition; psychological problems and environmental factors intensify these [46]. Conversely, optimal nutrition positively influences the quality of life and a sense of good health in the elderly [41]. EG significantly improved their nutritional status after 24 months of PA participation, showing a significant difference compared to the CG. Nutritional status is a multidimensional concept that includes, in addition to the indicators of nutritional health, psychological and social aspects of food. These aspects should be important for individuals to choose personal diet or enjoyment from their favorite food, eating with other persons, and sharing the pleasure of eating together. Moreover, each meal provides order and a sense of structure to the day of an older adult [47].

## 5. Conclusions

In the present long-term interventional study, PA was directly administered to the participants, closely monitoring intensity, volume, and correct technique execution. Consequently, the effects on the three indicators of successful aging (nutritional status, level of activities in daily life, and QoLP) might be attributed to this specific physical exercise protocol, punctually described in the methods. Moreover, the results were obtained from a randomized and controlled trial applied to a numerically significant sample (178 subjects). The combined aerobic and resistance training was the recommended protocol for the elderly by previous studies over time [18,48,49,50] and resulted in efficient improvements to nutritional behaviors and maintained the feeling of motor and cognitive competence [14,15]. The amount of food intake, as well as the components of the diet, could represent a warning bell to identify early psychological problems in the elderly [51]. The PA protocol performed in this study positively influenced the nutritional status of participants, favoring a good QoLP. Especially in the elderly, a good QoLP is closely related to general wellbeing linked to self-efficacy, autonomy, and frequent social interactions [52].

A major problem in modern societies is how to change the lifestyle and habits of older adults. PA programs must be efficient and prolonged such as to become an essential part of a daily routine. Moreover, to promote adherence to long-term PA programs, the protocols should ensure a suitable rate of exertion for the participants.

Aging is a normal physiological process but is really complex and is influenced by several factors that determine individuals’ lifestyles [53].

The amount of food intake, as well as the components of the diet, with the integration of moderate-to-high intensity PA into the daily routine, should favor the perception of successful aging.

## 6. Limitations

Several limitations of this study may be addressed: the nutritional status and the daily life management of the household, recreational, and working activities were assessed by questionnaires.

Significant differences in the age between the groups have been highlighted only after the dropout of some of the participants; however, this difference was only three years. Nevertheless, it may have influenced the results of the study.

The administered PA protocol was a conventional and validated protocol. Further studies will verify the results provided by innovative and more stimulating typologies of physical exercise such as PA in an enriched environment.

## Figures and Tables

**Table 1:** Sample characteristics.

Variable	*n*
**Experimental group**	90
**Age (mean ± SD)**	62.12 ± 7.88
**Gender**	
Male	32
Female	58
**PASE**	108.48 ± 45.21
**Control group**	88
**Age (mean ± SD)**	65.64 ± 8.12
**Gender**	
Male	28
Female	62
**PASE**	108.22 ± 47.04

PASE—“Physical activity scale for the elderly”: level of weekly physical activity performed at baseline.

**Table 2:** Results obtained by the two groups in the three tests.

Variable	Time	EG	CG	Significant Results
		Means ± SD	Means ± SD	
**SF-36**	**Baseline**	75.37 ± 17.51	73.52 ± 16.80 ^a^	^a^ the score of CG at 24 months was significantly lower compared to the CG baseline score (*p* = 0.033), ^b^ the score of CG at 24 months was significantly lower compared with EG at 24 months (*p* = 0.018).
	**6 months**	77.55 ± 15.80	74.22 ± 16.48	
	**12 months**	74.04 ± 17.58	70.79 ± 17.82	
	**24 months**	74.69 ± 16.06 ^b^	68.51 ± 17.25 ^a,b^	
**MNA**	**Baseline**	26.00 ± 2.90 ^c^	25.88 ± 2.79	^c^ the score of EG at 24 months was significantly higher compared to the EG baseline score (*p* = 0.003), ^d^ the score of EG at 24 months was significantly higher compared with CG at 24 months (*p* < 0.001).
	**6 months**	26.75 ± 2.64	26.74 ± 2.23	
	**12 months**	26.92 ± 2.23	26.44 ± 2.30	
	**24 months**	27.47 ± 1.82 ^c,d^	26.13 ± 2.44 ^d^	
**PASE**	**Baseline**	109.13 ± 45.03	107.93 ± 47.23 ^e^	^e^ the score of EG at 24 months was significantly higher compared to the EG baseline score (*p* = 0.008), ^f^ the score of CG at 12 months was significantly lower compared with CG baseline score (*p* = 0.011).
	**6 months**	112.59 ± 52.87	104.66 ± 44.65	
	**12 months**	106.02 ± 43.21	92.08 ± 41.97 ^e^	
	**24 months**	110.17 ± 39.40 ^f^	94.31 ± 41.97 ^f^	

EG = Experimental group; CG = Control Group; SF-36 = Short-Form 36-item; MNA = Mini nutritional assessment; PASE = Physical activity scale for the elderly.

## Data Availability

The data presented in this study are available on request from the corresponding author.
